# Poetry

**Published:** 1880-12

**Authors:** 


### SEPARATION.

A wall was grown up between the two—
A strong, thick wall, though all unseen—
None knew when the first stones were laid,
Nor how the wall was built, I ween.

And so their lives were wide apart,
Although they shared one board, one bed;
A careless eye saw naught amiss,
Yet each was to the other dead.

He, much absorbed in work and gain,
Grew soon unmindful of his loss;
A hard indifference worse than hate
Changed love's pure gold to worthless dross.

She suffered tortures all untold;
Too proud to mourn; too strong to die;
The wall pressed heavily on her heart:
Her white face showed her misery.

Such walls are growing day by day
'Twixt man and wife, 'twixt friend and friend;
Would they could know, who lightly build,
How sad and bitter is the end.

A careless word, an unkind thought,
A slight neglect, a taunting tone—
Such things as these, before we know,
Have laid the wall's foundation stone.

—*Springfield Republican.*

### ISOLATED.

We hold our dear ones with a firm, strong grasp,
We hear their voices, look into their eyes;
And yet, betwixt us in that clinging clasp
A distance lies.

We cannot know their hearts, howe'er we may
Mingle thought, aspiration, hope and prayer;
We cannot reach them, and in vain essay
To enter there.

Still, in each heart of hearts a hidden deep
Lies, never fathomed by its dearest, best.
With closest care our purest thoughts we keep,
And tenderest.

But, blessed thought! we shall not always so
In darkness and in sadness walk alone;
There comes a glorious day when we shall know
As we are known.

—*Elinor Gray, in the Atlantic.*

### ON THE EVE OF THE WEDDING.

O, love, before we part to-night,
Before the last "I will" is spoken—
Before the ring has touched my hand,
Of pure, true, endless love the token—
Before the Church with holy rite,
Her blessing on our love has given,
Look straight into my eyes with yours,
And answer me in sight of Heaven.

Is there within your heart of hearts
One lingering shadow of regret—
One thought that you have chosen ill?
Oh! speak—'tis not too late even yet.
Is there in all this world of ours,
One you have ever known or seen,
Whom, if you had earlier seen or known,
You would have crowned your chosen queen?

Is there? I pray you, tell me now,
And I will hold you bound no more.
I will not flinch to hear the truth.
It could not be so sad, so sore,
To know it now, as it would be
If by and by a shadow fell
Upon the sunshine of our home;
So, if you ever loved me—tell.

I'd hold you pure from blame, dear love;
And I would leave you free as air,
To woo and win that happier one;
All this for your dear sake I'd bear.
I will not say how I would pray
That God might have you in His care;
That would be easy—when I think
Of you, my heart is all one prayer.

But could I join her name with yours,
And call down blessing from above,
On her who had robbed me of my all—
My life—my light—my only love?
Yes! even that I'd try to do;
Although my lonely heart should break,
I'd try to say "God bless her!" too,
Through blinding tears, for your sweet sake.

I'm looking up into your eyes;
But though my own with tears are dim,
I read that in their true, clear depths,
Which tells me, "You may trust in him."
I will—I will!—It needs no words,
Though yours are flowing warm and fast,
And eloquent with truth and love,
Forgive my doubts—they are the last!

—*Chambers' Journal.*

### THE IDEAL.

I think the song that's sweetest
Is the one that's never sung;
That lies at the heart of the singer
Too grand for mortal tongue.
And sometimes in the silence
Between the day and night,
He fancies that its measures
Bid farewell to the light.

A picture that is fairer
Than all that have a part
Among the masterpieces
In the marble halls of art,
Is the one that haunts the painter
In all his golden dreams,
And to the painter only
A real picture seems.

The noblest, grandest poem
Lies not in blue and gold
Among the treasured volumes
That rosewood bookshelves hold;
But in bright, glowing visions,
It comes to the poet's brain,
And when he tries to grasp it
He finds his effort vain.

A fairy hand from dreamland
Beckons up here and there,
And when we strive to clasp it
It vanishes into air.
And thus our fair ideal
Floats always just before,
And we with longing spirits
Reach for it evermore.

